# Magnetic Field-Enhanced Agglutination Readout Combined With Isothermal Reverse Transcription Recombinase Polymerase Amplification for Rapid and Sensitive Molecular Detection of Dengue Virus

**DOI:** 10.3389/fchem.2021.817246

**Published:** 2022-01-24

**Authors:** Fanny Leon, Elena Pinchon, Charly Mayran, Aurélien Daynès, François Morvan, Jean-Pierre Molès, Jean-François Cantaloube, Chantal Fournier-Wirth

**Affiliations:** ^1^ Pathogénèse et Contrôle des Infections Chroniques et Emergentes, Université de Montpellier, Etablissement Français du Sang, Inserm, Université des Antilles, Montpellier, France; ^2^ HORIBA Medical, Montpellier, France; ^3^ Institut des Biomolecules Max Mousseron (IBMM), Université de Montpellier, CNRS, ENSCM, Montpellier, France

**Keywords:** innovative diagnostic, RT-RPA, magnetic field-enhanced agglutination, magnetic nanoparticles, dengue

## Abstract

Among the numerous molecular diagnostic methods, isothermal reverse transcription recombinase polymerase amplification (RT-RPA) is a simple method that has high sensitivity and avoids the use of expensive instruments. However, detection of amplified genomes often requires a fluorescence readout on costly readers or migration on a lateral flow strip with a subjective visual reading. Aiming to establish a new approach to rapidly and sensitively detect viruses, we combined RT-RPA with a magnetic field-enhanced agglutination (MFEA) assay and assessed the ability of this method to detect the dengue virus (DENV). Magnetization cycles accelerated the capture of amplified DENV genomes between functionalized magnetic nanoparticles by a fast chaining process to less than 5 min; the agglutination was quantified by simple turbidimetry. A total of 37 DENV RNA^+^ and 30 DENV RNA^−^ samples were evaluated with this combined method. The sensitivity and specificity were 89.19% (95% CI, 72.75–100.00%) and 100% (95% CI, 81.74–100.00%), respectively. This approach provides a solution for developing innovative diagnostic assays for the molecular detection of emerging infections.

## Introduction

The sensitivity and specificity of molecular methods used in laboratories and based on reverse transcription quantitative polymerase chain reaction (RT-qPCR) analysis for detection of viral RNA are excellent, but these methods have a long turnaround time and require trained personnel and costly equipment available only in major medical centers and not compatible with point-of-care testing. Hence the challenge is to develop innovative tests that combine the analytical performances of molecular approaches with the advantage of delocalized use. Rapid and simple diagnostic methods are fundamental for outbreak control, especially in endemic areas. Improving patient access to simple, fast molecular tests to expedite the diagnosis of patients who present to triage units or emergency units is urgently needed (Kelly-Cirino et al., 2019). Dengue, which we use here as a model, is a mosquito-borne disease that is considered to be the fastest spreading in the human world (Guzman et al., 2016; Musso et al., 2018). A recent study estimates that an average of 105 million dengue infections occur worldwide each year; this includes 51 million cases of febrile disease (Cattarino et al., 2020). Therefore, early detection at the infection’s acute phase is critical for improving epidemiological surveillance and management of disease (Campos et al., 2020). The disease is mainly caused by the four virus serotypes DENV-1, DENV-2, DENV-3 and DENV-4, which are antigenically and genetically related (Guzman et al., 2016). There are several methods aiming to simplify genomic amplification and detection that are currently being evaluated. In particular, reverse transcription loop-mediated isothermal amplification (RT-LAMP) assays have been established to detect the dengue virus (DENV) (Parida et al., 2005; Teoh et al., 2013; Kim et al., 2018; Lopez-Jimena et al., 2018). LAMP is a method of amplification that is specific and sensitive, but it requires using at least four different primer complexes designed to match all DENV strains circulating worldwide (Teoh et al., 2013; Lopez-Jimena et al., 2018). The recombinase polymerase amplification (RPA) assay is an alternative method of isothermal amplification that can detect nucleic acids (Piepenburg et al., 2006). With this technique, it is not necessary to melt the DNA in order that the primers are directed to the complementary target sequences. Instead, RPA enables DNA scanning and the exchange of the strands at cognate sites through the use of recombinase–primer complexes (Piepenburg et al., 2006; Li et al., 2018). A reverse transcription RPA (RT-RPA) assay developed and applied to DENV detection showed a good concordance with RT-qPCR assays. Moreover, this RT-RPA assay demonstrated various advantages versus RT-LAMP assays; these included an easy design of primers (only two for RT-RPA), a faster assay run time at a lower temperature, a higher sensitivity, and a relative ease of performance (Teoh et al., 2015; Xi et al., 2019; Xiong et al., 2020).

To scale down for applications at the point of care, we established a molecular assay to detect DENV-1–4 genomes by pairing an RT-RPA assay with a rapid readout by a magnetic field-enhanced agglutination (MFEA) assay. MFEA consists of generating magnetic fields with an electromagnet and applying these to reaction media to speed up capture of targets between magnetic nanoparticles (MNPs) through a rapid chaining process (Pinchon et al., 2020). Simple turbidimetry can analyze this agglutination in under 5 min (Pinchon et al., 2020; Leon et al., 2021). In this work, the targets represented by biotinylated RPA amplicons were captured between MNPs that had been grafted with specific anti-biotin antibodies and DENV probes. Here, we present the results of applying this rapid combined method to the molecular screening of DENV in human biological samples.

## Materials and Methods

### Viruses and Biological Samples

The French national arbovirus surveillance center (Centre National de Référence des arbovirus, CNR, Marseille, France) provided whole DENVs (DENV-1: Djibouti 2000 strain 1588; DENV-2: Martinique 1998 strain 703; DENV-3: Martinique 2001 strain 2023; DENV-4: Indonesia 1998 strain 812) as reference material. These were provided in frozen vials and contained ten-fold serial dilutions of infected cell culture supernatants (range 1,000–1 TCID_50_/ml). To control analytical specificity, we used two other cultivated whole arboviruses provided by the CNR, the Zika virus Asian lineage (ZIKV, French Polynesia 2013 strain) and the chikungunya virus (CHIKV, Reunion Island 2005 strain 6368), and one yellow fever virus strain derived from the vaccine (YFV). West Nile virus (WNV, Vircell, Granada, Spain) was derived from human samples. To study diagnostic performances, 37 DENV RNA^+^ samples extracted from DENV^+^ individuals were provided as frozen vials by the CNR. Levels of DENV RNA had been determined by RT-qPCR at the CNR. Negative controls were 30 plasma samples from individuals who had donated blood and who had no history of arbovirus contact (DENV RNA^−^ samples); these were provided by the French national blood service (Etablissement Français du Sang, EFS, Montpellier, France).

### Viral Nucleic Acid Extraction and One-Step RT-RPA Assay

The MagNA Pure Compact automated system alongside the MagNA Pure Compact Nucleic Acid Isolation Kit (Roche Diagnostics, Mannheim, Germany) was used (as per the manufacturer’s instructions) to extract viral nucleic acid. The input and elution volumes of the supernatants of infected cell cultures or biological samples were 200 and 50 μl, respectively. Viral nucleic acids that had been purified were aliquoted and stored at −80°C until needed. The entire process of automated extraction (30 min) and manual sampling (5 min) took 35 min. The design of the DENV primers required for RT-RPA was such that they targeted the highly conserved 3′-untranslated region (3′UTR) and was adapted from a previous publication (Abd El Wahed et al., 2015). The DENV-F forward primer (5′Biot- AAC AGC ATA TTG ACG CTG GGA GAG ACC AGA GAT C) was tagged with biotin at the 5’end. The labeled DENV-F and the DENV-R reverse primer (5’ ATT CAA CAG CAC CAT TCC ATT TTC TGG CGT TCT GTG), designed to amplify 97 bp of 3′UTR, were synthesized by Eurogentec (Seraing, Belgium). The RT-RPA assay was performed with a TwistAmp Basic kit (TwistDx, Cambridge, United Kingdom) supplemented with SuperScript II reverse transcriptase (Thermo Fisher Scientific, Waltham, Massachusetts, United States) to perform both reverse transcription (RT) and cDNA amplification in a single tube. The assay took place in a reaction volume of 50 μl that contained 5 µl of extracted DENV RNA. In brief, 29.5 µl of TwistAmp Rehydration buffer were combined with 2.4 μl of 5′biotinylated forward primer (10 μM), 2.4 μl of reverse primer (10 μM), 7.2 μl of DNase-free water, and 1 µl of SuperScript II (200 U). The reaction mixture (42.5 µl) was combined with the lyophilized RT-RPA enzyme mix and briefly mixed and spun. Then 5 µl of extracted DENV RNA were added to the reaction mixture and briefly mixed and spun. Finally, the addition of 2.5 μl of 280 mM magnesium acetate started the reaction. The tubes were put into a compact benchtop thermoblock (thermostat C, Eppendorf, Hambourg, Germany) at 42°C; they underwent incubation for 4 min followed by a brief mix and spin, and then were put back in the thermoblock for 26 min at 42°C. Each RT-RPA assay includes a negative plasma sample (Neg) and a no template control (Blank). After amplification, the products were analyzed directly using the MFEA assay or stored at −20°C until their use.

### Grafting Onto Magnetic Nanoparticles

A generic 15-mer tetrathiolated DENV DNA probe (5′TGG AAT GAT GCT GTA) aimed at detecting the 3′UTR of the four serotypes of DENV genomes was designed. We covalently grafted this DENV probe onto MNPs of a diameter of 200 nm (200 nm carboxyl-adembeads, Ademtech, Pessac, France) using an amino-polyethylene glycol-maleimide crosslinker according to our previous description (Pinchon et al., 2020). To achieve passivation of the MNPs, they were incubated with 1 ml of 1.5 M Tris-HCl, pH 8.8, for 20 min and 250 µl of a cysteine solution (80 mg/ml) for 10 min. The MNPs that had been covalently grafted with the DENV probe (MNP-Probes) were kept at 1% w/v in a dedicated buffer (10 mM glycine, 0.02% NaN_3_, 0.1% Synperonic F108 non-ionic surfactant, pH 9) at 4°C for ≤6 months.

Carbodiimide coupling chemistry was used to graft a second set of MNPs with anti-biotin (MNP-Abs); 10 µg of anti-biotin antibodies were added (Jackson Immunoresearch Europe Ltd, Cambridge, United Kingdom) to 1 mg of MNPs.

### Magnetic Field-Enhanced Agglutination Readout

Molecular detection was carried out in a disposable spectrophotometric cuvette; an electromagnet providing a magnetic field of 15 mT surrounded the cuvette. A photodiode and an LED source emitting at 650 nm performed the simple optical detection (Pinchon et al., 2020). The MFEA readout consists of application of a magnetic field to the reaction medium in order to speed up capture of DNA targets between MNP-Probes and MNP-Abs. Three magnetization (60 s) and relaxation (30 s) cycles resulted in aggregate formation by the fast chaining process. The total variation of the optical density at 650 nm (ΔOD_650nm_) measured prior to and after three magnetization cycles was used to express the turbidity signal. The time required for detection was under 5 min. Measurements were carried out in duplicate.

To determine the limit of detection (LOD), synthetic 15-mer DENV DNA oligonucleotides (5′TAC AGC ATC ATT CCA) biotinylated at their 5′-end (Eurogentec, Angers, France) and complementary to the tetrathiolated DENV probe were serially diluted two-fold from 1,000 to 0.98 pM in the hybridization buffer (HB, 6X SSPE, 5X Denhardt solution). For the MFEA readout, incubation of 160 µl of oligonucleotides with 3 µl of MNP-Probes (1% w/v) occurred under agitation for 5 min at 37°C. Next, the mix was added to two disposable cuvettes that contained 1.5 µl of MNP-Abs (1% w/v) and 72.5 µl of mix in order to carry out duplicate measurements. The LOD was established by determining the mean value of a no template control (Blank) plus the standard deviation multiplied by three.

### Detection of Amplified DENV Genomes

**FIGURE 1:**
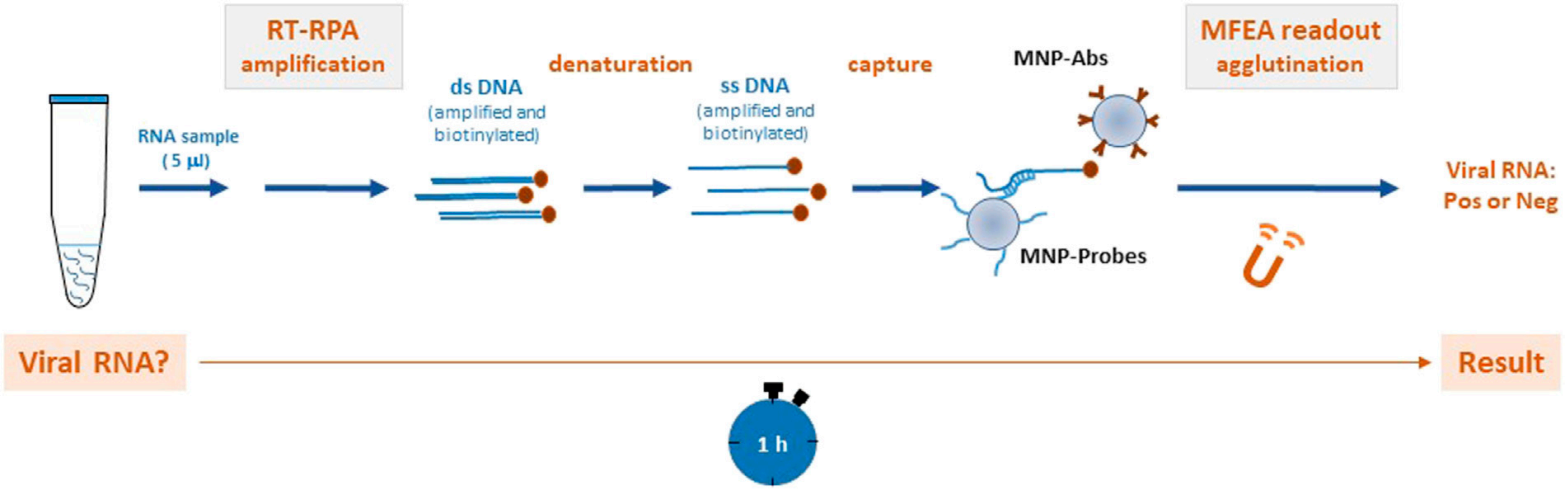
Method for the rapid molecular detection of the dengue virus (DENV) based on reverse transcription recombinase polymerase amplification (RT-RPA) combined with a magnetic field-enhanced agglutination (MFEA) readout DENV RNA is amplified using the RT-RPA method. After a denaturation step, the biotinylated DENV products are captured between magnetic nanoparticles (MNPs) that have been grafted with specific anti-biotin antibodies (MNP-Abs) and DENV tetrathiolated DNA probes (MNP-Probes). MFEA accelerates the capture of the targets by a fast chaining process.

RT-RPA–amplified DENV DNAs were diluted 1:10 in HB and denatured at 95°C for 10 min in order to generate single-stranded biotinylated DNA; subsequently, this was incubated with MNP-Probes under agitation for 5 min at 37°C. Next, the mix was added to two disposable cuvettes and MNP-Abs were added to perform the MFEA assay as described on Figure 1. In each assay, synthetic 15-mer DENV DNA oligonucleotides biotinylated at their 5′-end were used at 1,000 pM as positive controls. Blank samples were defined as those that contained HB, MNP-Probes, MNP-Abs, and RT-RPA mix without DENV genome.

### Statistical Analysis

Table 1 details the 95% confidence intervals (CI) [calculated as per Newcombe’s method (Newcombe, 1998)]. GraphPad Prism 8.0 software was used to generate scatterplots and receiver operating characteristic (ROC) curves. ROC curves show the performance of the test as the true-positive fractions (% sensitivity) compared with the false-positive fractions (100—% specificity).

**TABLE 1:** Molecular MFEA readout on biological samples.

**Sample type**	**Samples, *n***	**Samples correctly detected, *n***	**Diagnostic sensitivity^a^, % (95%CI)**	**Diagnostic specificity^b^, % (95% CI)**	**Accuracy^c^, %**
DENV	37	33	89.19 (72.25–100.00)	—	94.37
Healthy	30	30	—	100.00 (81.84–100.00)	

Supplementary Table S1 describes the detailed results. CI, confidence interval; DENV, dengue virus; MFEA, magnetic field-enhanced agglutination.

a [number of positive samples/(number of positive samples + number of false-negative samples)] × 100.

b [number of negative samples/(number of negative samples + number of false-positive samples)] × 100.

c [(number of negative samples + number of positive samples)/(number of negative samples + number of positive samples + number of false-negative samples + number of false-positive samples)] × 100.

## Results

### Combining RT-RPA Amplification With MFEA Readout

**FIGURE 2:**
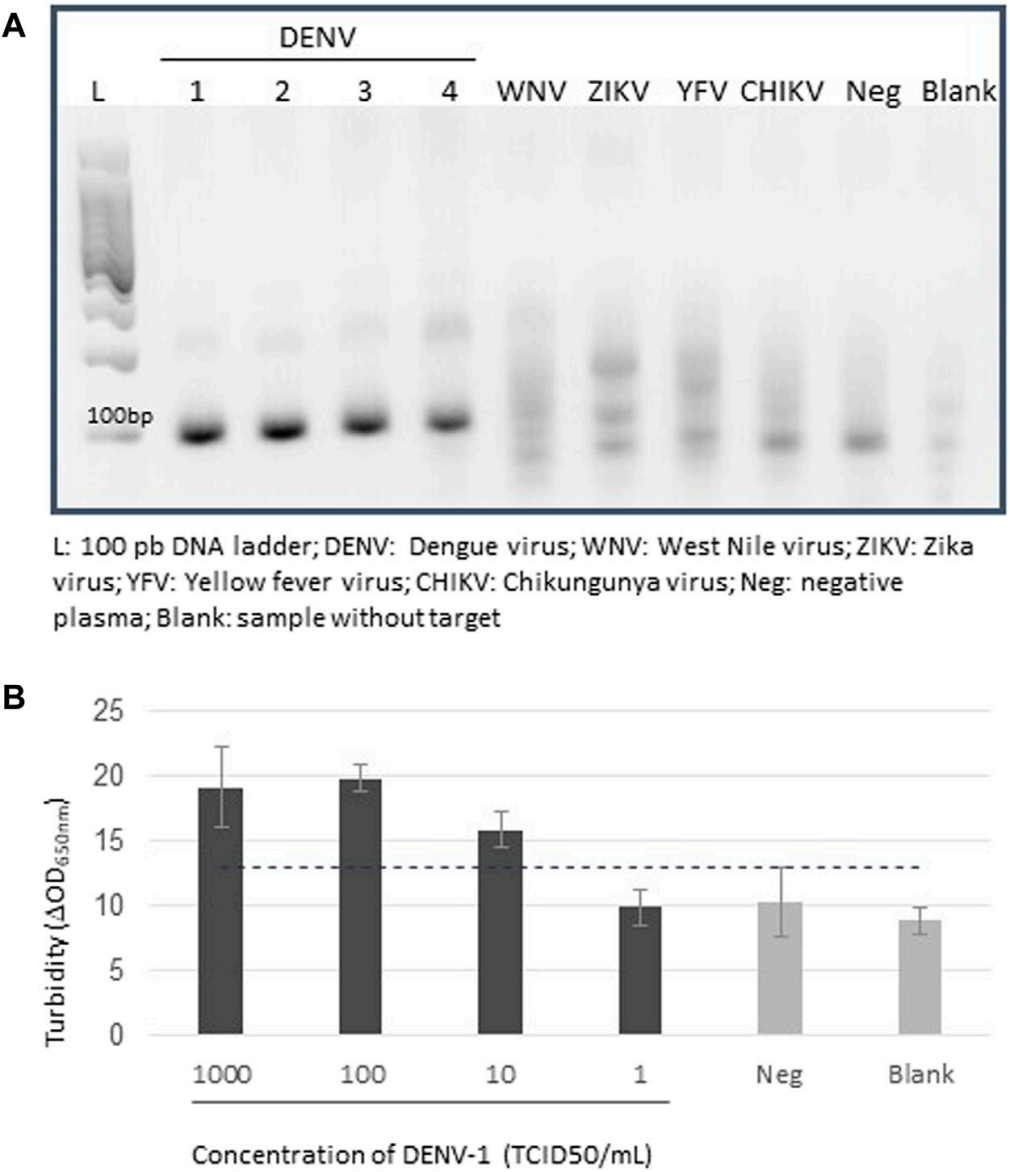
Detection of amplified DENV genomes. **(A)** Electrophoresis of RT-RPA–amplified products on a 2% agarose gel. The four serotypes of dengue viruses were tested (DENV-1–4). The West Nile virus (WNV), Zika virus (ZIKV), chikungunya virus (CHIKV), and yellow fever virus (YFV) were used as specificity controls. Negative plasma samples (Neg) were plasma samples from blood donors. No template control was defined as a blank sample (Blank). **(B)** Detection of amplified DENV-1 genomes. Analysis took place on serial dilutions from 1,000 to 1 TCID_50_/ml of DENV^+^ cell culture supernatants, each tested in three replicates. Negative plasma samples (Neg) were plasma samples from individuals who had donated blood. Following extraction and RT-RPA, DENV genomes were analyzed using MFEA readout. No template control was defined as blank sample (Blank). Error bars show standard deviation of triplicate measurements.

Our detection step using MFEA and turbidity measurement was first controlled on synthetic DENV sequences that were captured on MNP-Probes. The turbidity variation (ΔOD_650nm_) increased with the concentration of synthetic DNA and showed a LOD of 7.81 pM (Supplementary Figure S1). RT-RPA successfully amplified DENV-1–4 samples (Figure 2A). The RT-RPA assay did not amplify the closely related arboviruses, which were from the *Flavivirus* genus (WNV, ZIKV, YFV) or the *Alphavirus* genus (CHIKV). We used serial dilutions from 1000 to 1 TCID_50_/ml of whole DENVs, with serotype 1 as a model, to examine the combined approach’s analytical sensitivity. The LOD was 10 TCID_50_/ml (Figure 2B), a value comparable to fluorescence assays performed previously after RT-PCR amplification of DENV RNAs (Pinchon et al., 2020), indicating that the combination of RT-RPA and MFEA is able to detect DENV with a high sensitivity. Including all steps from mix preparation to the end of incubation, the RT-RPA assay had a mean turnaround time of 40 min, compared with a turnaround time of more than 2.5 h when an RT-PCR amplification step is used (Leon et al., 2021). The MFEA readout was performed in less than 5 min. Altogether the assay, including amplification by RT-RPA and detection of amplified products by MFEA, took 1 h.

### Detection of DENV in Biological Samples

**FIGURE 3:**
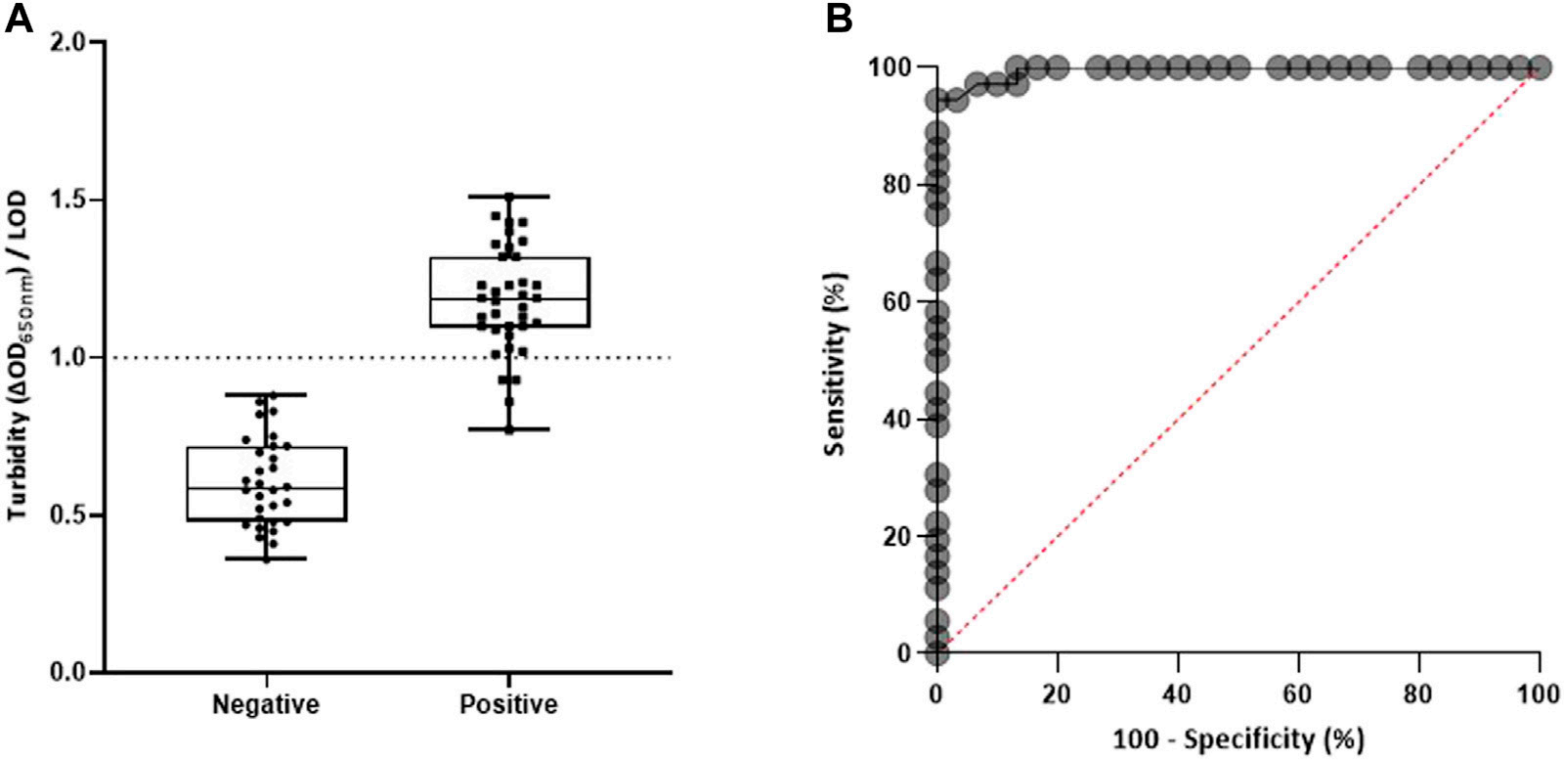
Molecular MFEA readout for DENV RNA^−^ and DENV RNA^+^ plasma samples. **(A)** DENV RNA^−^ samples (*n* = 30) and DENV RNA^+^ samples (*n* = 37) were evaluated. The difference in optical density at 650 nm (ΔOD_650nm_) measured prior to and following three magnetization cycles was used to express the turbidity signal. The LOD (limit of detection) is the mean value of blank samples plus three standard deviations. Individual points on the scatterplot are the turbidity/LOD signal ratio calculated by the molecular MFEA readout for a sample. Data are expressed as median ratios with interquartile ranges. *p* value <0.0001; unpaired *t*-test. **(B)** Receiver operating characteristic (ROC) curve. The ROC curve is generated by plotting true-positive fractions (sensitivity %) (true-positive samples/true-positive plus false-negative samples) versus false-positive fractions (100 − specificity %) (false-positive samples/false-positive plus true-negative samples). AUC, area under the ROC curve.

Figure 3 reveals the potential of molecular MFEA readout to discriminate between DENV^+^ and DENV^–^ human plasma samples. Out of 37 DENV^+^ samples, four samples detected positive with the reference RT-qPCR method were not detected using our combined approach. Two of them were samples with low DENV viral loads (Supplementary Table S1; cycle threshold >30). The two others corresponded to DENV samples of serotype 4. The ROC curve analysis revealed that there was excellent discrimination between DENV^+^ and DENV^–^plasma samples; the area under the ROC curve was 0.9949 (95% CI, 0.9851–1.00). In total, the MFEA readout identified 33 of 37 DENV^+^ samples (Table 1) (89.19% diagnostic sensitivity; 95% CI, 72.75–100.00%). A 94.37% accuracy value confirmed the ability of the MFEA readout to accurately differentiate between DENV^+^ and DENV^−^ samples. No signal was observed in DENV^−^ plasma samples (100% diagnostic specificity; 95% CI, 81.74–100.00%).

## Discussion

The key lesson from previous outbreaks, such as yellow fever or Ebola in Africa or the recent Zika and COVID-19 pandemics, is that to rapidly contain outbreaks, diagnostic tests that can easily be used in the field are extremely important (Kelly-Cirino et al., 2019). Quickly recognizing viremic patients is extremely useful in terms of patient care and medical treatment decisions and also in terms of addressing public health control measures. The early diagnosis of dengue has been reported using commercial rapid diagnostic tests for the immunocapture in serum of the non-structural viral protein NS1 (Matusali et al., 2020; Aik et al., 2021). These immunochromatographic strip assays, presented in a lateral flow cassette, are sensitive and easy to perform, but a lack of specificity can be observed due to cross reactions with other arboviruses such as the Zika or chikungunya viruses (Aik et al., 2021). In addition, differences between the structures of NS1 in the various dengue serotypes could be an issue in diagnosis. Finally, the visual reading of these colorimetric rapid tests can still be subjective even for trained personnel (Yow et al., 2021). The detection of viral genomes provides evidence of infection, but typical real-time PCR methods are not appropriate when screening on site.

Here, we have developed a new molecular strategy for detection of viral RNA during an infection’s acute phase; the approach uses isothermal RT-RPA requiring a simple thermoblock combined with rapid optical detection based on MFEA. This method still has limitations: i) like all other molecular techniques, it comes after the pre-analytical step; ii) it does not allow multiplexing in this format; iii) RPA is available as a commercial kit at TwistDX, the single manufacturer on the market. Improvements to replace the automated extraction step by rapid lysis protocols are in progress, but our combined method is a proof-of-concept for developing simple sample-to-answer molecular diagnostics. The RT-RPA assay means that thermal cycling devices are not required, and the assay provides very efficient amplification. The easy-to-use detection in the homogenous phase by turbidimetry avoids the fluorescence detection or subjective visual reading of lateral flow strips previously reported (Abd El Wahed et al., 2015; Teoh et al., 2015; Li et al., 2018; Xi et al., 2019; Xiong et al., 2020). Applied to the detection of DENV, this combined approach shows good limits of detection—7.81 pM on synthetic single-stranded DENV DNA and 10 TCID_50_/mL on cultivated whole DENVs—and a good specificity. These analytical performances are relevant by comparison with DNA hybridization based assays previously published (Supplementary Table S2). Our results suggest that the amplification of DENV-4 on agarose electrophoresis appears less efficient than that of DENV-1–3. This observation correlates with the fact that the design of the RT-RPA primers was initially focused to amplify DENV-1–3 (Abd El Wahed et al., 2015). In addition, potential mismatch between the DENV-4 amplified product because of sequence variability within the 3′UTR and the generic tetrathiolated DENV probe could also explain the lack of detection of two DENV- 4 samples in the DENV^+^ panel. However, the design of the tetrathiolated probes and primers can easily be adapted and could be improved to detect all DENV serotypes and variants or, in contrast, could be extended to include four different tetrathiolated DENV probes, each specific for one serotype, to perform fast genotyping of viruses (Lereau et al., 2013). The use of RT-RPA here required 40 min for the amplification of viral RNA compared with the more than 2 hours using RT-PCR observed in a previous study (Leon et al., 2021). Its combination with the simple and very fast MFEA readout opens the way for the development of a sensitive testing system that is easy to use and that can be delocalized within the field for molecular detection of emerging infections.

## Data Availability

The original contributions presented in the study are included in the article/Supplementary Material, further inquiries can be directed to the corresponding author.

## Abbreviations

RT-RPA, reverse transcription recombinase polymerase amplification; MNP, magnetic nanoparticles; MFEA, magnetic field-enhanced agglutination; RT-qPCR, reverse transcription quantitative polymerase chain reaction; Ct, cycle threshold.
