# Supplementary material for: Magnetic Field-Enhanced Agglutination Readout Combined With Isothermal Reverse Transcription Recombinase Polymerase Amplification for Rapid and Sensitive Molecular Detection of Dengue Virus
Source: Front Chem. 2022 Jan 24;9:817246. doi: 10.3389/fchem.2021.817246 (PMC8819590; doi:10.3389/fchem.2021.817246)
Supplement: Supplementary file 1 [file DataSheet1.pdf]

SUPPLEMENTAL TABLE 1: Full data set of DENV+ clinical samples used in the molecular MFEA readout

Ct, cycle threshold; RT-qPCR, reverse transcription quantitative polymerase chain reaction; RT-RPA, reverse transcription recombinase polymerase amplification

Serotype	Samples	RT-qPCR (Ct value)	RT-RPA MFEA
DENV-1	1	28	+
	2	9	+
	3	12	+
	4	8	+
	5	11	+
	6	19	+
	7	27	+
	8	14	+
	9	13	+
	10	9	+
	11	25	+
	12	28	+
	13	31	+
	14	13	+
	15	29	+
	16	19	+
	17	33	-
	18	18	+
DENV-2	19	10	+
	20	12	+
	21	16	+
	22	14	+
	23	14	+
	24	16	+
DENV-3	25	18	+
	26	26	+
	27	19	+
	28	25	+
	29	17	+
	30	33	-
	31	23	+
DENV-4	32	14	+
	33	19	-
	34	10	+
	35	27	+
	36	11	+
	37	13	-
Total	37	/	33

SUPPLEMENTAL FIGURE 1: Detection of synthetic DENV DNA sequence
Signals are measured for DENV sequences (dark) complementary to the DENV probe grafted onto magnetic nanoparticles. Dashed line indicates the limit of detection, taken as the mean value of blank samples (Blank) plus three standard deviations. Each point represents a measurement performed in duplicate. Error bars indicate the standard deviation of two independent measurements for each point.

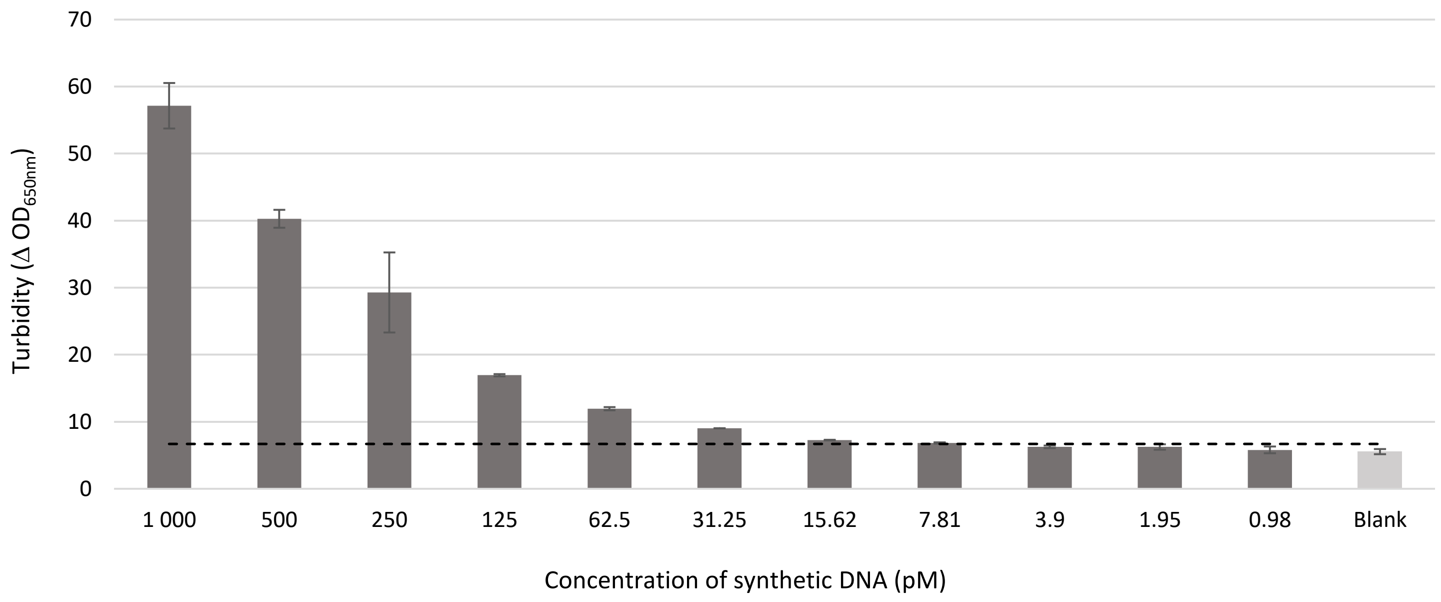

SUPPLEMENTAL TABLE 2: DNA hybridization based assays for the detection of DENV

Assay	Detected analyte	Amplification method	Detection method / Detection time (min)	Limit of detection	Reference
Electrochemical sensors	Synthetic DENV DNA	No	Differential Pulse Voltammetry	3 nM	Oliveira et al., 2015
Electrochemical sensors	Synthetic DENV DNA	No	Amperommetry	17 nM	Odeh et al., 2017
Electrochemical sensors	Synthetic DENV DNA	No	Cyclic Voltammetry, Differential Pulse Voltammetry , Electrochemical Spectroscopy	120 zM	Tripathy et al., 2017
qRT-PCR TaqMan probes	Synthetic DNA	One step qRT –PCR	Real time fluorescence (65 min)	7900-37000 c/mL	Waggoner et al., 2016
qRT-PCR TaqMan probes	In vitro transcribed RNA	One step qRT –PCR	Real time fluorescence (80 min)	3000 genome equivalent/mL	Simmons et al., 2016
RT-LAMP	DENV RNA	DENV RT LAMP 4 primers	Visualization under UV Illumination 365 nm (5 min)	3500 c/ml	Kim et al., 2018
RT-LAMP	DENV RNA	DENV RT LAMP 9 primers	Visual observation of fluorescence Under UV light	100-RNA copies/test	Teoh et al., 2013
RT-LAMP	DENV RNA	DENV RT LAMP 4 mixes 3-14 primers	Fluorescence scanner	100-1000 RNA molecules/test	Lopez-Jimena et al., 2018
MNPs capture + fluorescence	Synthetic DNA	No	Time-Resolved fluorescence 610 nm (DELFIA europium) (180 min)	0,1 pM	Leon et al., 2019
MNPS capture + magnetic field enhanced agglutination	DENV RNA	One step RT-PCR 2 primers	Turbidity 650 nm (5 min)	100 $TCID_{50}$/mL	Leon et al., 2020
MNPs capture + magnetic field enhanced agglutination	Synthetic DNA	No	Turbidity 650 nm (5 min)	7,81 pM	This study - Leon et al.,
MNPs capture + magnetic field enhanced agglutination	DENV RNA	One step Isothermal RT-RPA 2 primers	Turbidity 650 nm (5 min)	10 $TCID_{50}$/mL	This study - Leon et al.,

Supplemental references

Oliveira, N., Souza, E., Ferreira, D., Zanforlin, D., Bezerra, W., Borba, M. A., Arruda, M.,
Lopes, K., Nascimento, G., Martins, D., Cordeiro, M. and Lima-Filho, J. (2015). A Sensitive and Selective Label-Free Electrochemical DNA Biosensor for the Detection of Specific Dengue Virus Serotype 3 Sequences. *Sensors*, *15*, 15562-15577.

Odeh, A. A., Al-Douri, Y., Voon, C. H., Mat Ayoub, R., Gopinath, S. C. B., Odeh, R. A., Ameri,
M., Bouhemadou, A. (2017). A needle-like Cu2CdSnS4 alloy nanostructure-based integrated electrochemical biosensor for detecting the DNA of Dengue serotype 2. *Microchimica Acta*, 184, 2211-2218.

Tripathy, S., Vanjari, S. R. K., Singh, V., Swaminathan, S., Singh, S. G. (2017).
Electrospun manganese (III) oxide nanofiber based electrochemical DNA nanobiosensor for zeptomolar detection of dengue consensus primer. *Biosens Bioelectron*, 90, 378–387.

Waggoner, J. J., Ballesteros, G., Gresh, L., Mohamed-Hadley, A., Tellez, Y., Sahoo, M. K.,
Abeynayake, J., Balmaseda, A., Harris, E., Pinsky, B. A. (2016). Clinical evaluation of a single-reaction real-time RT-PCR for pan-dengue and chikungunya virus detection. *J Clin Virol.*, 78, 57–61.

Simmons, M., Myers, T., Guevara, C., Jungkind, D., Williams, M., Houng H. S. (2016).
Development and Validation of a Quantitative, One-Step, Multiplex, Real-Time Reverse Transcriptase PCR Assay for Detection of Dengue and Chikungunya Viruses. *J Clin Microbiol.*, 54, 1766-1773.

Leon, F., Meyer, A., Reynier, R., Blanc, E., Bruyère-Ostells L., Brès, J. C., Simonin, Y.,Salinas, S., Gallian, P., Leparc-Goffart, I., Biron, A., Dupont-Rouzeyrol, M., Morvan, F., Vasseur, J. J., Foulongne, V., Van de Perre, P., Cantaloube, J. F., Fournier-Wirth, C. (2019). An Innovative Multiplexed and Flexible Molecular Approach for the Differential Detection of Arboviruses. *J Mol Diagn*., 21, 81-88.
